# Kinetics and Spatial Distribution of β‑Sheet Development in TDP‑43_CTD_ Condensate Maturation

**DOI:** 10.1021/acschemneuro.6c00226

**Published:** 2026-05-22

**Authors:** Sashary Ramos, Matthew D. Watson, Jennifer C. Lee

**Affiliations:** Laboratory of Protein Conformation and Dynamics, Biochemistry and Biophysics Center, National Heart, Lung, and Blood Institute, 369964 National Institutes of Health, Bethesda, Maryland 20892, United States

**Keywords:** amyotrophic lateral sclerosis, phase separation, secondary structure, Raman microspectroscopy, amide-I, amide-III, bend-libration

## Abstract

Cytosolic inclusions of aggregated TAR DNA-binding protein 43 (TDP-43) are hallmarks of neurodegenerative disorders such as amyotrophic lateral sclerosis and frontotemporal lobar dementia. A prevailing hypothesis suggests that TDP-43 condensates undergo a liquid-to-solid transition during maturation, involving the formation of β-sheet–rich, amyloid-like aggregates. To test this hypothesis, we sought to study the temporal and spatial evolution of protein secondary structure within individual condensates by Raman spectroscopy. We measured *in vitro* β-sheet development of the C-terminal domain of TDP-43 (TDP-43_CTD_) at the single-condensate level under physiological solution conditions. All condensates showed apparent single-exponential kinetics (*k* = 1.6 × 10^–5^ s^–1^) for the disordered-to-β-sheet transformation, as indicated by increased amide-I intensity and a shift of the amide-III band to lower energy. Interestingly, the water bend-libration band exhibited a slower rate (*k* = 4.0 × 10^–6^ s^–1^), suggesting that changes in the water environment lag behind protein conformational rearrangement. Further, Raman maps revealed that protein density is highest near the condensate center, whereas β-sheet content is mostly uniform in the interior of the condensate. The unexpected difference between the spatial distributions of β-sheet content and protein density challenges the typical concentration-dependent model of protein aggregation. Importantly, rare events were captured where condensates exhibited spatially asymmetric β-sheet development, revealing localized structural heterogeneity not detectable by ensemble measurements. Collectively, these results provide insight into the temporal and spatial dynamics of protein structure within TDP-43_CTD_ condensates and demonstrate the utility of Raman spectral imaging for tracking condensate maturation.

## Graphical abstract

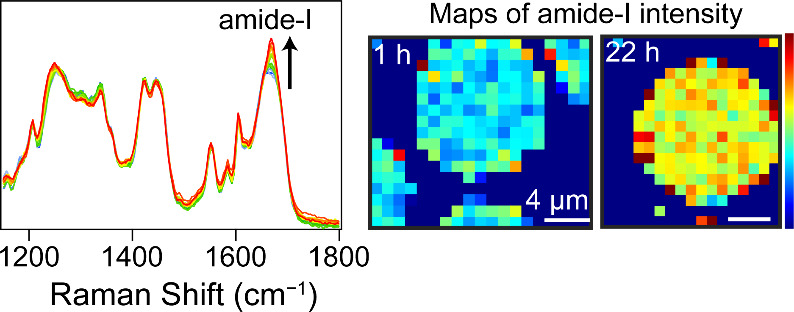

## Introduction

TAR DNA-binding protein 43 (TDP-43) is a ubiquitously expressed protein that belongs to the heterogeneous ribonucleoprotein family.^1^ Primarily located in the nucleus, TDP-43 plays a central role in RNA metabolism, processing, and transport.^1,2^ Importantly, cytosolic TDP-43 inclusions are the pathological hallmarks of amyotrophic lateral sclerosis (ALS) and frontotemporal lobar dementia (FTLD).^3,4^ Although TDP-43 aggregates have traditionally been characterized as amorphous, recent cryoelectron microscopic studies have found TDP-43 amyloid fibrils in patients.^5–7^ Importantly, fibril structures determined from *ex vivo* patient materials comprise similar fragments (residues 282–360,^5^ 272–360,^6^ and 284–345 ^7^) of the C-terminal domain (CTD) of TDP-43 (TDP-43_CTD_). While distinct from the fibril structures determined from *ex vivo* materials, both recombinant full-length TDP-43 ^8,9^ and TDP-43_CTD_ ^10^ also form amyloids with β-sheet-containing cores composed of CTD residues (residues 279–360,^8^ 304–348,^9^ and 276–414 ^10^), highlighting the importance of the CTD in the aggregation process of TDP-43. In addition, co-occurrence of TDP-43 pathology has been documented in Alzheimer’s disease, dementia with Lewy bodies, Parkinson’s, Huntington’s, and chronic traumatic encephalopathy,^11^ prompting further interest in the broader role of TDP-43 aggregation in neurodegeneration.^12^

TDP-43 is a 414-amino acid protein (Figure 1A, gray) composed of an N-terminal domain (NTD), two RNA-recognition motifs (RRM1 and RRM2), and an intrinsically disordered CTD (Figure 1A, blue, residues 274–414). In the literature, the CTD is also referred to as a prion-like domain (PrLD) or a low complexity domain (LCD) because the sequence is enriched in amino acids (Gly, Ser, Asn, Ala, and Gln) found in prion domains that drive their amyloid formation.^13,14^ Notably, the majority (∼75%) of ALS-related mutations are found in the CTD,^15–17^ further substantiating it as a pertinent region for study. A characteristic related to its function is the participation of TDP-43 in the formation of biomolecular condensates, such as stress granules (SGs) and nuclear stress bodies.^18,19^ The underlying mechanism of SGs formation is liquid–liquid phase separation (LLPS), in which TDP-43 and other RNA-binding proteins (e.g., Ras GTPase-activating protein-binding protein 1 (G3BP-1), fused in sarcoma (FUS), and heterogeneous nuclear ribonucleoprotein A1 (hnRNPA1))^20,21^ and nucleotides sequester from the cellular milieu into liquid-like droplets, facilitated by transient, multivalent interactions (e.g., π-π, electrostatics, and π-cation interactions^22–25^ ). SGs have been hypothesized to act as foci for aggregation of amyloidogenic proteins by creating local areas of dramatically increased protein concentration under prolonged cellular stress.^26,27^ In fact, *in vitro* and *in cellulo* experiments have shown that TDP-43_CTD_ is essential for TDP-43 phase separation and is highly aggregation prone.^28–32^ Thus, it is crucial to understand the nature and environment of secondary structure transition from disordered-to-β-sheet conformation within TDP-43_CTD_ condensates as they age.

**Figure 1:**
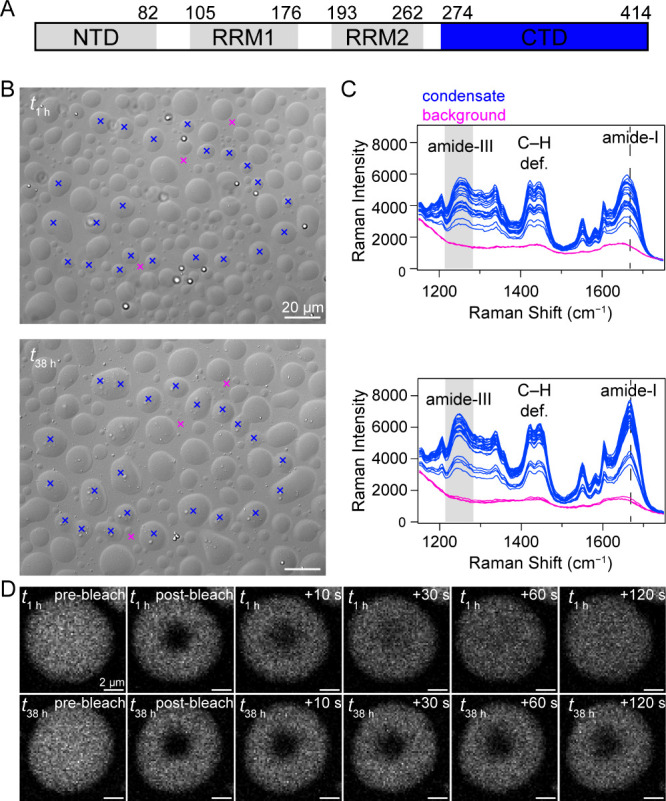
Raman spectral imaging of TDP-43_CTD_ condensates. (A) Schematic representation of the polypeptide regions within the TDP-43 sequence: N-terminal domain (NTD), RNA recognition motif (RRM), and C-terminal domain (CTD, residues 274–414, construct used in this study). (B) DIC images taken at 1 and 38 h after deposition on the cover glass. Additional time points are shown in Figure S1. [CTD] = 15 μM in 10 mM NaPi, 200 mM NaCl, pH 7.4, 20 °C. Scale bars are 20 μm. Locations (×) where Raman spectra were collected for condensates (blue, *n* = 22) and background (magenta, *n* = 3). (C) Corresponding Raman spectra collected at 1 h (top) and 38 h (bottom) for condensates (blue) and background (magenta). Amide-I peak position (dashed line), C–H deformation, and amide-III band region (shaded area) are indicated. Full spectra are shown in Figure S2. (D) Representative fluorescence recovery after photobleaching image sequences taken at 1 and 38 h after deposition on the cover glass. [CTD] = 15 μM with 0.1 mol % Alexa Fluor 532-labeled monomer in 10 mM NaPi, 200 mM NaCl, pH 7.4, 20 °C. Scale bars are 2 μm.

Various biophysical techniques, including fluorescence, nuclear magnetic resonance (NMR), atomic force microscopy (AFM), and vibrational spectroscopies have been used to study TDP-43_CTD_ phase separation and amyloid formation *in vitro*.^28,30,33–42^ These studies have primarily relied on ensemble-averaged techniques and do not distinguish the individual behavior of condensates, reporting only on average characteristics. Additionally, some of these approaches do not directly report on protein secondary structural changes. To fully understand the role of TDP-43_CTD_ condensate formation in protein aggregation, methods capable of directly probing secondary structure at the single condensate level in aqueous environments are necessary, as demonstrated in Raman-based studies of model systems^43^ and the ALS-related protein FUS.^44–46^

In prior work, we coupled Raman spectroscopy with an inverted microscope (Raman microspectroscopy) to spatially resolve the formation of β-sheet-containing amyloid-like structures upon the liquid-to-solid transition of TDP-43_CTD_ condensates.^47^ Raman spectroscopy is a vibrational scattering technique that measures intrinsic molecular vibrations; protein secondary structure can be determined by analysis of the Raman-active amide-I and amide-III bands.^48,49^ The amide-I band primarily reports on C=O stretching, while the amide-III vibration reports on the in-phase contribution of C–N stretching and N–H in-plane bending of the peptide backbone.^48,50–52^ In particular, amyloid β-sheet structure exhibits a narrow and intense amide-I peak and a shift in the amide-III band to lower energy.^53^ While both amide bands report on β-sheet content, the amide-III band has been shown to be more sensitive to fibril structural differences (polymorphism),^52,54^ as it has been suggested to report on tertiary structure (i.e., β-sheet packing).

Here, we sought to advance our understanding of the relationship between LLPS and amyloid formation by resolving individual condensates spatially and temporally. We tracked individual TDP-43_CTD_ droplets with Raman microspectroscopy to evaluate the kinetics of β-sheet development and used Raman spectral imaging (RSI) to generate Raman maps that aid in visualizing spatial heterogeneity of protein density, β-sheet content, and water environments within condensates. While protein density maps show a circular pattern highlighting increased density in the center, β-sheet content and water maps are more nuanced, suggesting distinct distributions. This work provides a detailed, label-free view of how molecular structure and spatial organization evolve during TDP-43_CTD_ condensate aging, offering new insight into the pathways linking phase separation and pathological aggregation.

## Results

### Tracking Individual TDP-43_CTD_ Condensates by Raman Spectroscopy

To probe morphological changes in TDP-43_CTD_ condensates and molecular differences within them over time, we utilized a home-built inverted Raman microscope.^54^ Our CTD construct constitutes residues 274–414, which was produced by TEV cleavage removal of N-terminal Thio6 and hexa-histidine tags, which were used for expression and affinity purification, respectively. Individual frozen aliquots were thawed and desalted into water immediately prior to measurements. LLPS was initiated *via* a salt jump to achieve physiologically relevant solution conditions: 10 mM sodium phosphate and 200 mM NaCl, pH 7.4. Protein condensates were allowed to settle for 1 h (*t*_1 h_) before data acquisition. At each time point, a differential interference contrast (DIC) image (Figure 1B) was taken, and a Raman spectrum (Figure 1C) was collected at the center of each individual condensate (*n* = 22). The same condensates were probed over time. At *t*_1 h_, a varied size distribution of condensates is observed, with most appearing as symmetrical droplets (Figure 1B, *top*). The condensates are fluid at early time points, as evidenced by the readily observed fusion events (Figure S1A), whereas at later times, the aged condensates no longer change in shape (Figure S1B), suggesting solidification. To confirm the changes in fluidity, fluorescence recovery after photobleaching (FRAP) experiments were performed under identical conditions with the addition of 0.1 mol % Alexa Fluor 532-labeled TDP-43_CTD_ (Figure 1D). Droplets are highly fluid at *t*_1 h_, demonstrated by essentially complete fluorescence recovery within ∼1 min, whereas aged droplets harden significantly at *t*_38 h_, with minimal recovery even after several minutes.

To obtain consistent time-course spectral data, first, a *z*-step scan was performed on an arbitrary droplet (Figure S3), and the maximum protein signal (i.e., C–H deformation) was used to determine the Raman imaging plane of the condensates. At each subsequent time point, the same *z*-position was maintained by matching the O–H stretch intensity (3430 cm^–1^) of the buffer background. At *t*_1 h_, the Raman spectra of TDP-43_CTD_ (Figure 1C, *top*) show key protein signatures including contributions from the amide-III (1220–1291 cm^–1^), C–H deformations (1395–1486 cm^–1^), and the amide-I (1627–1710 cm^–1^). At this early time point, the amide-I band, which reports on secondary structure, appears broad, suggesting that the proteins are largely disordered after droplet deposition, consistent with previous work on a mutant variant.^47^ After 38 h of aging (Figure 1C, *bottom*), the amide-I peak sharpens with a maximum at ∼1669 cm^–1^, and the amide-III band shifts to lower energy, indicating β-sheet structure has developed within the condensates, as reported previously.^47^ These spectral features for TDP-43_CTD_ fibrils are similar to other amyloids such as α-synuclein and Aβ_1–40._ ^53^ To corroborate the presence of amyloid-like structures, we used thioflavin-T (ThT), an amyloid-sensitive fluorophore,^55^ and performed a fluorescence imaging time course, which clearly show the enhancement of ThT fluorescence intensity as the condensates age (Figure S4).

### Kinetics of β-Sheet Development in Condensates

To evaluate the temporal evolution of protein conformational changes in a single condensate, background spectra were subtracted from the sample spectra, and the resulting Raman spectra were normalized by protein concentration using the integrated area of the C–H deformation bands. Representative Raman spectra of a single condensate recorded at different aging times are shown in Figure 2A. Time-dependent enrichment of β-sheet structure in a single condensate is indicated by the narrowing and increased intensity of the amide-I peak (dashed line) and red-shifting of the amide-III band (gray) in accord with Figure 1C. Here, with the removal of bulk water contributions *via* background subtraction, changes in the high-frequency N–H stretch region are revealed. However, these spectral differences are convoluted with the overlapping O–H stretch, which also appears to be changing with time.

**Figure 2:**
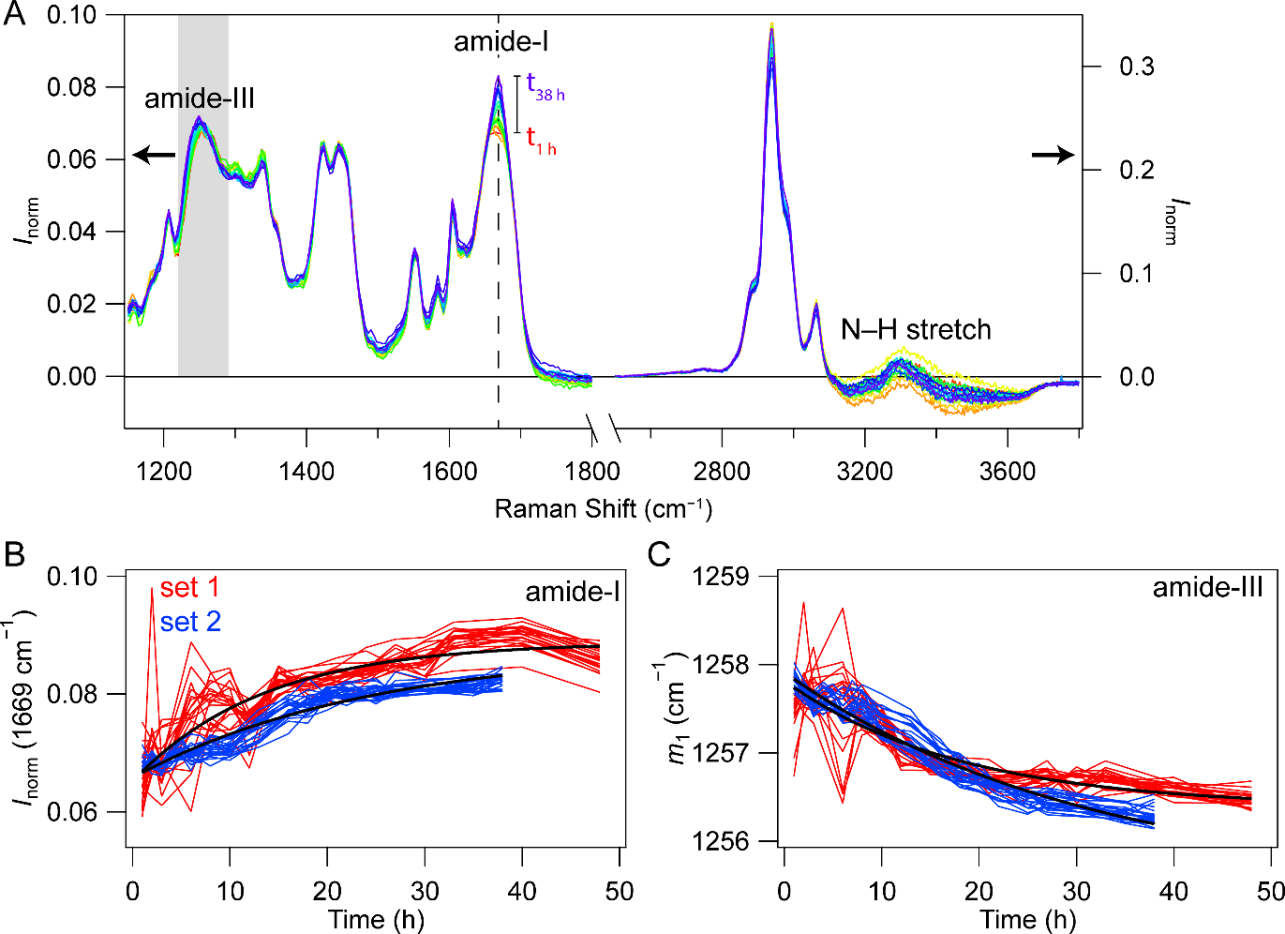
Analysis of Raman spectral changes associated with TDP-43_CTD_ condensate aging. (A) Representative set of time-dependent Raman spectra of a single condensate. Background subtraction and normalization to the integrated area of the C–H deformation have been performed. Amide-I peak position (dashed line), amide-III band region (gray), and N–H stretch are indicated. [CTD] = 15 μM in 10 mM NaPi, 200 mM NaCl, pH 7.4, 20 °C. Intensity scales for low and high frequencies are shown on the left and right axes, respectively. (B) Kinetics of the normalized amide-I band intensity at 1669 cm^–1^ for individual condensates (*k* = 1.6 ± 0.6 × 10^–5^ s^–1^). (C) Kinetics of the weighted mean frequency (first moment, *m*_1_) of the amide-III band calculated between 1220–1291 cm^–1^ for individual condensates (*k* = 1.6 ± 0.1 × 10^–5^ s^–1^). Two independent data sets (*n* = 22 (set 1, red) and *n* = 21 (set 2, blue)) are shown. Global fits for each set are shown as black lines; rate (*k*) constants are reported as averages and standard deviations from both sets.

Kinetics of β-sheet development were quantified by the changes in amide-I band intensity (Figure 2B) and the frequency shift in the amide-III band (weighted mean frequency or first moment, *m*_1_, Figure 2C). Two independent experiments using different biological replicates were measured and analyzed (*n* = 43 condensates). The first (red) and second (blue) sets contained 22 and 21 condensates, respectively. Kinetics of the amide bands for both sets are highly consistent with each other and sufficiently described by single exponential functions, yielding similar rate constants of 1.6 × 10^–5^ s^–1^.

### Raman Maps of Protein Density and β-Sheet Content in Condensates

Next, Raman maps were collected for individual condensates at various time points (1, 6, 13, 22, and 38 h) to obtain spatial information on protein distribution and secondary structure content within a condensate (Figure 3A–E). Here, a full Raman spectrum is collected at 1 μm steps, and a Raman map is generated for frequencies of interest: protein density (C–H deformation, Figure 3 *top row*), β-sheet content (amide-I intensity, Figure 3 *second row*), tertiary structure (*m*_1_ of amide-III, Figure 3 *third row*), and water (*m*_2_ of bend-libration, Figure 3 *bottom row*). Protein density maps show radial symmetry with higher protein density at the center of the condensate (Figure 3, *top row*). To aid visualization, an intensity profile through the center line (white dashed line) of the condensate is shown above each map. Clearly, the highest protein concentration sits at the center of the droplet and decreases outwardly. This profile is similar for condensates at different aging times. Strikingly, the normalized spatial distributions (intensity profiles) of β-sheet content and tertiary structure are more uniformly distributed throughout the droplets. It is notable that there are hotspots or larger variations at the periphery of the droplets beginning at 6 h (Figure 3B), which are not reported by the intensity profile analysis. This observation is more apparent in maps of β-sheet content than tertiary structure. Overall, the progression of these maps is consistent with the kinetics data shown in Figure 2A and B, with increased intensity of the amide-I band and the shift of the amide-III *m*_1_ value to lower frequency over time. A second set of condensate maps is shown in Figure S5.

**Figure 3:**
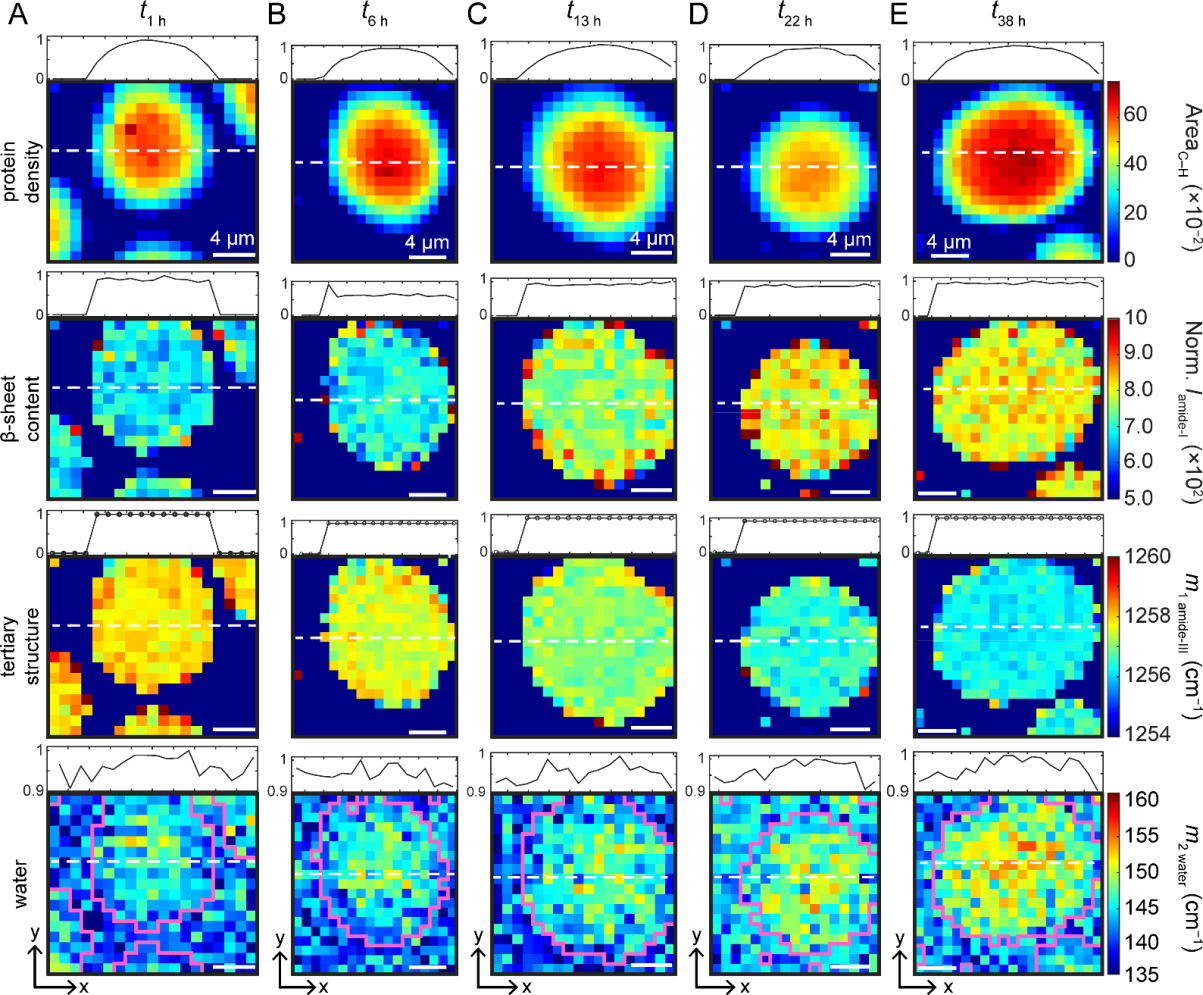
Raman maps of individual TDP-43_CTD_ condensates. Maps were generated by using the integrated area of the C–H deformation (1395–1486 cm^–1^) for protein density, the normalized amide-I band intensity at 1669 cm^–1^ for β-sheet content, the weighted mean frequency (first moment, *m*_1_) of the amide-III region (1220–1291 cm^–1^) for tertiary structure, as well as the spectral variance (second moment, *m*_2_) of the bend-libration (1900–2450 cm^–1^) for water at (A) *t*_1 h_, (B) *t*_6 h_, (C) *t*_13 h_, (D) *t*_22 h_, and (E) *t*_38 h_. Intensity profile taken at the center line of the condensate (white dotted line) is shown above each map. Condensate locations are outlined in pink as a guide to the eye (bottom row). The water map was generated from uncorrected spectra, whereas background-corrected spectra were used to generate the other maps. [CTD] = 15 μM in 10 mM NaPi, 200 mM NaCl, pH 7.4, 20 °C. Step size was 1 μm. Scale bars are 4 μm. Additional condensate maps are shown in Figure S5.

### Evolution of Water in Condensates

To further understand the environment inside the protein condensates, Raman maps were generated by utilizing the spectral variance (second moment, *m*_2_) of the bend-libration of water (Figures 3A–E, *bottom row*), a water vibration which has been demonstrated to be sensitive to water associated with biomolecules.^56^ The spectral variance reports on the heterogeneity of the water population, where a larger spectral variance suggests a greater population of states, reflecting differences in the local environment. Overall, increased spectral variance is observed closer to the center of the condensate, reminiscent of the protein density maps. While the intensity profiles at the center line of the condensates are noisier, they show a maximal value at the center. Interestingly, the spectral variance increase spreads outward as the condensate ages. These changes appear at later times compared to the secondary structural changes. This delayed trend of increasing water spectral variance over time is also corroborated by the kinetics obtained for individual droplets, which yielded a slower rate constant of 4.0 ± 0.3 × 10^–6^ s^–1^ (Figure S6).

### Cross-Sectional View of a Protein Aggregate

In both sets of experiments, few condensates developed an amyloid-like or filamentous morphology that may be on pathway to amyloid formation. To investigate the molecular composition of one of these converting condensates at *t*_46 h_ (Figure 4A), Raman data were collected from a slice across the condensate along the *x*-*z* plane. Raman maps of protein density (Figure 4B) and β-sheet content (Figure 4C) were generated as described above. The protein density shows one condensate sitting on the coverslip on the left side, with a second feature on the right, higher up, away from the coverslip. This is interesting because it indicates that an aggregate extends from another area and is unlikely to have formed at the coverslip. Similar to the *x*-*y* plane images (Figure 3, *second row*), β-sheet content appears to be more intense at the periphery (i.e., apical), whereas most of the protein density is shown to be in the central slices of the condensate (Figure 3, *top row*).

**Figure 4:**
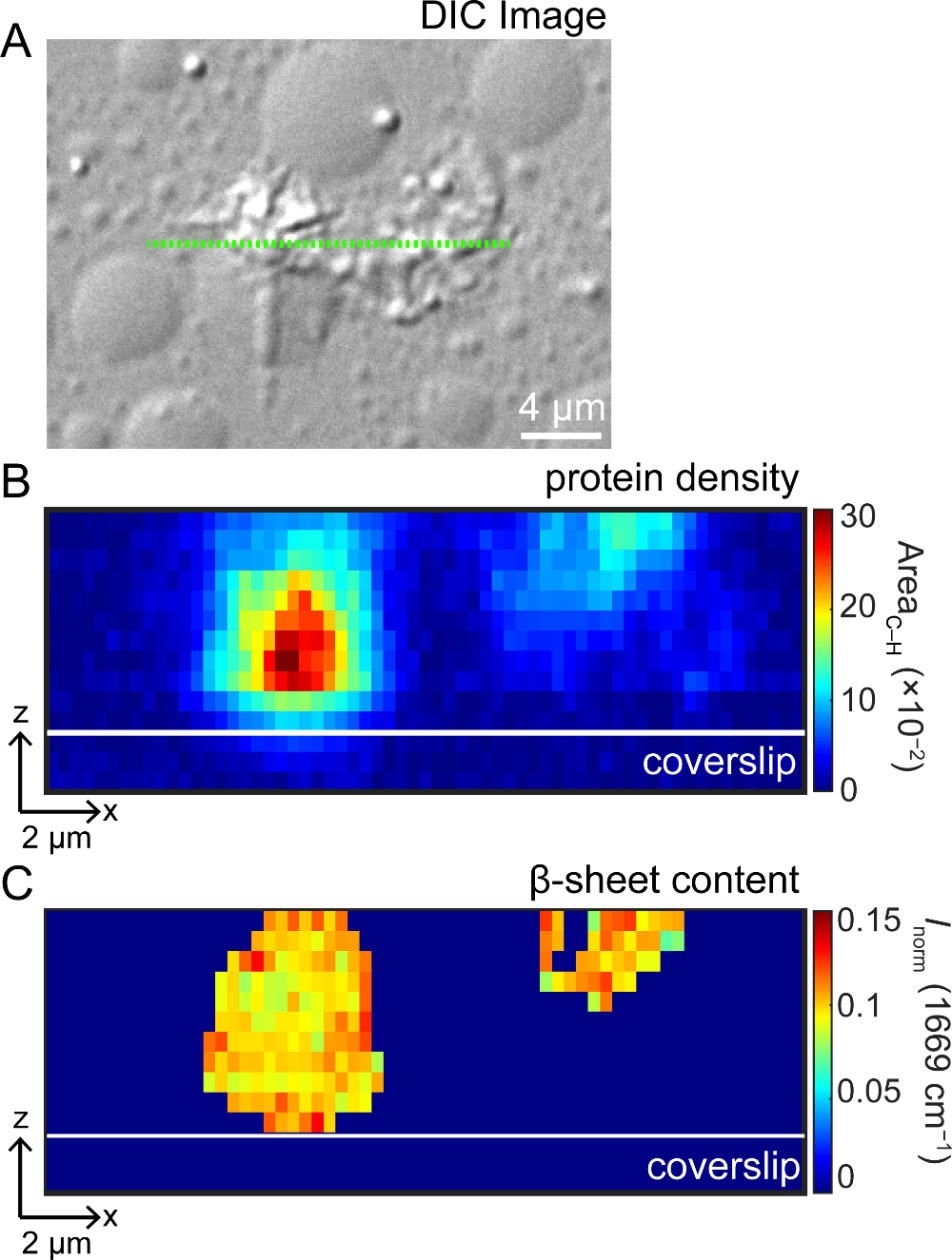
Horizontal *z*-slice of a condensate with aggregate-like morphology. (A) DIC image of the protein condensate at *t*_46 h_ showing morphological changes. Green line designates the slice where Raman data were collected at various *z*-positions. *Z*-slices of (B) the integrated area of the C–H deformation (1395–1486 cm^–1^) depicting the protein density and (C) the normalized amide-I band intensity at 1669 cm^–1^ showing β-sheet content. [CTD] = 15 μM in 10 mM NaPi, 200 mM NaCl, pH 7.4, 20 °C. Step size in the *x*-direction was 0.3 and 0.5 μm in the *z*-direction. Scale bars are 2 μm. Positions of the coverslip are as indicated.

### Conformational Heterogeneity within a TDP-43_CTD_ Condensate

Another converting condensate was observed at *t*_23 h_ (Figure S7). Maps of protein density and β-sheet content are shown in Figure 5A and B, respectively. Similar to other single plane maps (Figure 3, *top row*), the protein density is located in the center of the droplet. However, the β-sheet content shows an asymmetric distribution, with increased β-sheet near the edge of the left upper quadrant, unlike that observed in the other condensates. The same condensate was mapped again at *t*_39 h,_ and interestingly, the β-sheet content has spread along the upper edge and toward the middle of the condensate. To verify the differences in secondary structure, a 3 × 3-pixel region-of-interest (ROI) was selected at two distinct locations within the condensate. ROI 1 (r_1_) is located in the top left quadrant of the condensate, while ROI 2 (r_2_) is located at the bottom right quadrant. The average spectrum of each ROI is shown in Figure 5C. Comparing spectra of the two ROIs confirms that a more intense amide-I band at 1669 cm^–1^ is associated with r_1_, and that over 16 h, the amide-I intensity continues to increase in r_1_, whereas no changes were observed for r_2_ (Figure S8).

**Figure 5:**
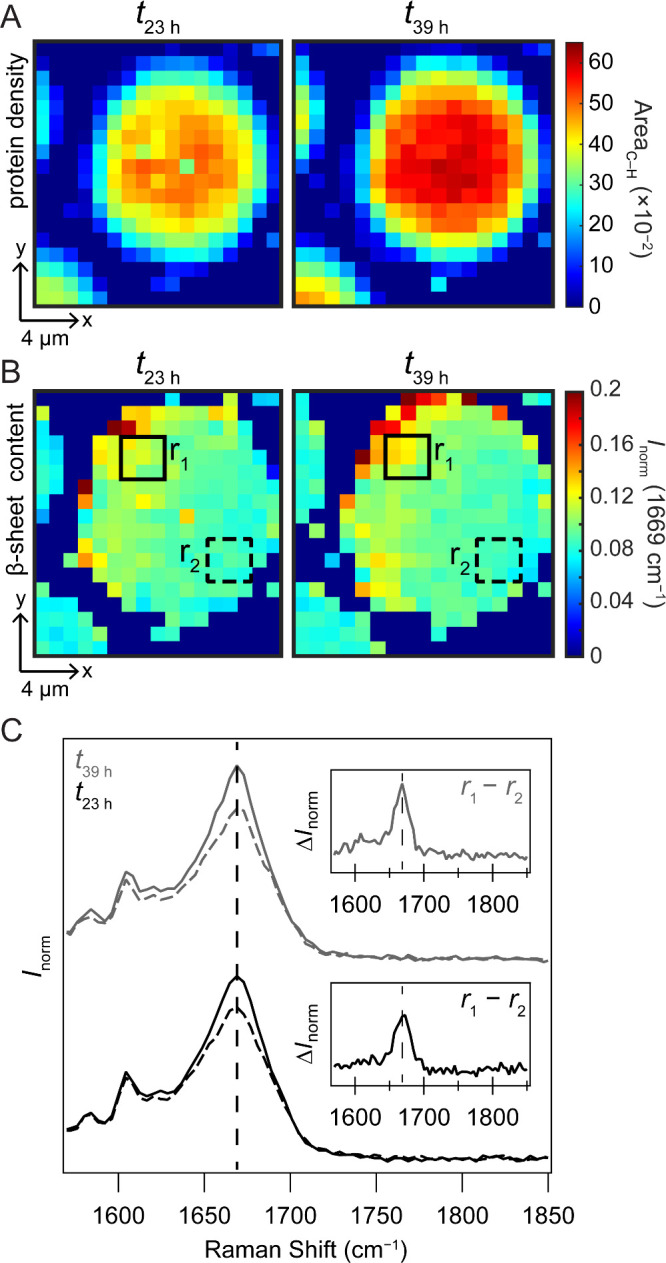
Spatial heterogeneity of protein secondary structure within a single condensate at different time points. Maps were generated by using (A) the area of the C–H deformation calculated from 1395–1486 cm^–1^ and (B) the normalized amide-I band intensity at 1669 cm^–1^. Aging times are as indicated. Step size is 1 μm. Scale bars are 4 μm. (C) Spectra averaged from the indicated regions-of-interest (r_1_ (solid) and r_2_ (dashed)) in panel A after 23 (black) and 39 h (gray) of aging. Insets show the difference spectra comparing r_1_ and r_2_ (r_1_(*t*_23 h_)–r_2_(*t*_23 h_), black and r_1_(*t*_39 h_)–r_2_(*t*_39 h_), gray). At both time points, r_1_ contains a higher β-sheet content compared to that of r_2_. Dashed lines indicate the frequency position of 1669 cm^–1^. [CTD] = 15 μM in 10 mM NaPi, 200 mM NaCl, pH 7.4, 20 °C.

## Discussion

The evolution of individual protein condensates has not been fully explored; however, understanding this process is crucial for uncovering the factors that determine the fate of proteins in what was once a one-phase homogeneous mixture. We employed Raman microspectroscopy to individually track the evolution of 43 condensates to provide spatially resolved insight into condensates undergoing structural conversion.

DIC imaging enabled us to observe the fusion and growth of TDP-43_CTD_ condensates over the course of the experiment (Figure 1B), while RSI provided information on spectral signatures associated with protein density and conformational development. Importantly, by normalizing spectra to the C–H deformation band, we were able to decouple changes in protein concentration from those in conformation, thereby allowing direct comparison of structural evolution across time points and condensates. At the spectral level, sharpening of the amide-I band at 1669 cm^–1^ and a red-shift in the amide-III band (Figure 1C and D) are consistent with an increase in β-sheet content as the condensates aged. The development of a more ordered secondary structure with aging suggests condensates serve as environments that promote dynamic, time-dependent structural rearrangement rather than static disordered states.

A quantitative analysis of the amide-I and -III bands revealed that both spectral features report on β-sheet development with similar kinetics (Figure 2B and C). The determined rate constants are an effective average over the individual condensates, akin to an ensemble measurement. Interestingly, although secondary structural changes similar to amyloid structures are observed, the apparent single-exponential β-sheet development suggests a rate-limiting conformational transition occurs during droplet maturation. This is unlike the conventional nucleation-polymerization model for amyloid formation, which exhibits sigmoidal behavior with a prolonged lag phase and rapid growth. Under our solution conditions, amyloid formation is a rare event and challenging to capture in a single field of view. Interestingly, a similar observation was made for hnRNPA1 mutants, where β-sheet content was observed in condensates with FTIR spectroscopy, but no evidence of fibrillar material was seen.^57^

Raman maps of TDP-43_CTD_ condensates suggest protein density is not uniform (Figure 3, *top row*), with higher concentrations observed near the center of the condensate. It remains to be determined how much surface wetting plays a role and whether protein density distribution would be the same in a freely diffusing droplet. Interestingly, the β-sheet content did not mirror the pattern of the protein density. The normalized Raman maps generated for the amide-I and -III bands (Figure 3, *second* and *third rows*) showed a more even distribution of β-sheet structure throughout most condensates at each time point, whereas the protein abundance is most concentrated in the center and decreases near the edges. Profiles taken near the centers of the amide-I and amide-III maps further highlight that secondary structural changes are distributed uniformly throughout the condensate slice. This observation suggests that β-sheet formation within condensates is not driven solely by local protein concentration; rather, the condensate environment as a whole influences this conformational rearrangement. This finding supports a more nuanced relationship among packing, conformation, and dynamics in phase-separated systems, rather than a simplistic, concentration-focused view, an observation that has also been made in cellular studies of stress granules.^58,59^

Analysis of the spectral variance of the water bend-libration revealed spatial and temporal heterogeneity in the water environment within condensates. Water maps (Figure 3, *bottom row*) show a larger spectral variance in the center of the condensate, which spreads toward the edge as the condensate ages. This does not mirror the protein density or β-sheet maps exactly, but does indicate a higher spectral variance is associated with the same area as increased protein density, an observation which may be correlated but not causative. Kinetics of the variance of the bend-libration were also obtained (Figure S6) and showed a slower rate than that of the secondary structure development, indicating that solvent dynamics are not strictly concomitant. These results suggest that the condensate environment is a complex landscape in which water molecules experience increasingly diverse environments as protein packing increases. Given the calculated rates, it can be inferred that protein dynamics may modulate the water environment. The lack of a direct spatial correlation between water and β-sheet content suggests that water contributions alone are insufficient to explain the structural changes within a condensate.

Under our conditions, most condensates displayed a comparable increase in β-sheet content; however, a very small subset underwent conversion to amyloid-like or filamentous material (Figure S7). Raman maps of these events (Figure 5) revealed spatial heterogeneity in β-sheet content that was not observed in other condensates. In particular, an asymmetric increase in amide-I intensity over time indicated localized β-sheet formation followed by amyloid propagation. This propagation event appears to extend from the periphery of the condensate. It is possible that a surface defect or preformed aggregate could have induced this transition, as can be inferred from the *z*-slice image shown in Figure 4, where a part of the aggregate is not touching the cover glass surface or a condensate. It remains unclear what causes this heterogeneity in secondary-structure development or what determines the fate of distinct droplets.

In prior TDP-43_CTD_ studies, distinct amyloid initiation sites on aging condensates have been observed. Babinchak *et al*.^30^ suggest fibrils emanate from within droplets based on AFM images, whereas amyloid aggregates were visualized by Laurents *et al*.^35^ to be enriched at the condensate surface using ThT.^55^ Notably, work on FUS has suggested a core–shell model for maturing condensates based on coherent anti-Stokes Raman scattering (CARS) microscopy, where the entire periphery of the condensate has a distinct, homogeneous β-sheet secondary shell compared to that of its unstructured core.^44^ Herein, our results suggest a more nuanced scenario. First, amyloid-like β-sheet structure is homogeneously distributed throughout all aged condensates, unlike the core–shell condensates proposed for FUS,^46,60^ and they do not readily transform into fibrillar morphology. Rather, fibrillar aggregates are a rare occurrence. Moreover, when fibrillar aggregates are observed, the transitioning condensates have a heterogeneous distribution of β-sheet structure with enrichment localized at certain points along the periphery. Thus, these findings do not support the ideas of TDP-43_CTD_ aggregation arising purely from the interior of the condensate or the surface. Clearly, more work is needed to elucidate the interplay between condensate maturation and amyloid formation of TDP43_CTD_. For example, a construct and/or solution conditions that promote fibril formation will enable the simultaneous tracking of condensate aging and fibril development within a single field of view. Additionally, future experiments using full-length TDP-43 and incorporating relevant biomacromolecules, such as RNA, will be valuable.^61^

In conclusion, Raman microspectroscopy was used to monitor the molecular changes of TDP43_CTD_ condensates over time. Tracking of individual protein condensates revealed little kinetic heterogeneity between independent condensates, with all tracked condensates appearing to develop β-sheet structure at a similar rate. Mapping of individual condensates showed a mostly random distribution of β-sheet content, with amide-I/-III maps revealing differences at the periphery not observed in the protein density maps. Additionally, in rare instances, there were observations of β-sheet-rich regions that propagated to other areas of the condensate over time, resembling amyloid propagation. The ability to capture these rare events highlights the importance of single-condensate studies with spatial resolution. An ensemble measurement would miss such an event, masking spatial nuances of aggregation pathways. Taken together, our results show that Raman microspectroscopy is a valuable tool for characterizing individual biomolecular condensates and offers new mechanistic insights into their conformational changes during aging.

## Experimental Procedures

### Reagents

Chemicals were obtained from Sigma unless otherwise specified.

### Protein Expression and Purification

TDP-43_CTD_ (residues 274–414) was expressed in LB media as previously described.^39^ The construct consisted of an N-terminal Thio6 expression sequence (MGSDKI), hexa-histidine tag, and TEV cleavage site (ENLYFQ) followed by residues 274–414. This was cloned into a pET21a­(+) plasmid using NdeI/XhoI restriction sites by GenScript (pET21a­(+)-ThioHisTEV-hTARDBP­(Δ1–273)). Protein was purified as previously described with some modifications.^39^ Briefly, cell pellets (3 g) were resuspended in 50 mL lysis buffer (40 mM Tris, 0.5 M NaCl, 1 mM EDTA, 1 mM PMSF, pH 8), lysed by sonication on ice using 1/2” tip (setting 7, 50% duty cycle for 10 min) and a Branson Sonifier 450. Inclusion bodies were collected by centrifugation (38,700 × *g* for 25 min at 4 °C) and resolubilized overnight in 50 mL denaturing buffer (20 mM Tris, 0.5 M NaCl, 7 M Urea, 20 mM imidazole, 1 mM PMSF, pH 8) with a rotary mixer at 4 °C. Soluble protein fraction was clarified by centrifugation (38,700 × *g* for 25 min at 4 °C) and purified using a HisPrep FF 16/10 column (GE Healthcare) equilibrated with denaturing buffer. TDP-43_CTD_ was eluted with a linear gradient of imidazole, pooled, and buffer exchanged into TEV cleavage buffer (20 mM Tris, 1 M GuHCl, pH 8) using a HiPrep desalting 26/10 column (GE Healthcare). TEV cleavage reaction was performed by adding 10,000 U TEV (GenScript) and 1 mM DTT to the protein solution (70–100 μM, determined on a Cary 300 Series UV–Vis Spectrometer (Agilent Technologies) using molar absorptivity at 280 nm (ε_280 nm_ = 19,480 M^–1^ cm^–1^ based on amino acid content)) at 4 °C. After 90 h of incubation, cleaved protein was isolated by passing through a HisPrep FF 16/10 column (GE Healthcare). A final step of purification was achieved using a Mono S 10/100 GL column (GE Healthcare) equilibrated with low ionic strength buffer (20 mM MES, 7 M Urea, pH 6). Protein was eluted with a linear gradient of NaCl. Purified protein was concentrated using an Amicon stirred ultrafiltration cell and a MWCO 3kD filter (Millipore) to 150–230 μM, aliquoted, flash frozen using liquid N_2,_ and stored at –80 °C until use. Protein homogeneity and identity were evaluated by LC-MS (NHLBI Analytical Biochemistry Core). Measured mass was 13680.8 Da (Calculated 13680.58). All buffers were filtered (0.22 μm) and stored at 4 °C.

### Fluorescent Labeling of Proteins

TDP-43_CTD_ was desalted into denaturing buffer (20 mM Tris, 500 mM NaCl, 20 mM imidazole, 7 M urea, pH 8) on a PD10 desalting column (Cytiva), then into H_2_O on a second PD10 desalting column. The labeling reaction was initiated by mixing TDP-43_CTD_ (43 μM) with Alexa Fluor 532 NHS ester (Thermo) in a dye:protein ratio of 3.75:1. The reaction was heated intermittently in a 100 °C heat block to prevent droplet formation. After 1 h the protein was diluted with an equal volume of GuHCl (8 M) and desalted into storage buffer (20 mM MES, 7 M urea, pH 6.0) on a PD10 desalting column. The protein was concentrated in an Amicon centrifugal filter unit (3 kDa MWCO), diluted with GuHCl (8 M) and again desalted into storage buffer (20 mM MES, 7 M urea, pH 6.0) on a PD10 desalting column. The protein and dye concentrations were determined from the absorbances at 280 and 532 nm based on ε_280 nm_ = 17,990 M^–1^ cm^–1^ and ε_532 nm_ = 81,000 M^–1^ cm^–1^ and a correction factor of 0.09 at 280 nm. Labeling was quantitative.

### TDP-43_CTD_ Condensate Formation

Samples were prepared as previously reported, with some modifications.^47^ Briefly, protein was thawed at RT (20–21 °C) and buffer exchanged into deionized water using Micro Bio-Spin 6 columns (Bio-Rad) following manufacturer instructions. Protein concentrations were determined by UV–visible spectroscopy (ε_280 nm_ = 17,990 M^–1^ cm^–1^ based on amino acid content). Phase separation was initiated by a salt jump using a 10× buffer stock (100 mM NaPi, 2 M NaCl, pH 7.4), with a final protein concentration of 15 μM.

### Raman Spectral Imaging and Data Analysis

Samples (50 μL) were imaged in a sealed, 18-well chambered cover glass with #1.5 high-performance cover glass (CellVis, C18–1.5H) using a home-built inverted Raman microscope as previously described^62^ with some modifications. The iHR320 spectrometer and Symphony II BIDD CDD Detector (Horiba) were controlled using LabSpec 6.7.1 software. Briefly, 10 mW of the 514 nm line of an argon-ion laser (CVI Melles Griot, 35-MAP-431–200) was used as the excitation source, and beam collimation and expansion were achieved using a ZBE1A beam expander (Thorlabs). A 60× oil objective (UPLAPO60XOHR 1.5 NA, Olympus) and a 600 mm^–1^ grating were used. The CCD image was binned from 124 to 132 pixels in the *y*-dimension (20 kHz rep rate and best dynamic range gain was used for individual spectrum collection, whereas 1 MHz rep rate and best dynamic range gain was used for map collection). DIC images were collected using an Infinity 3–6 URM CCD (Teledyne Lumenera). Individual droplets were collected with 2 × 10-s accumulations. Droplet maps were collected in 1-μm steps with 1-s accumulation time at each pixel. Raman data were processed using LabSpec 6.7.1 and Matlab 2024b (Mathworks). Raman spectra were corrected using a polynomial calibration generated from well-defined bands of cyclohexane.^63^ First moment of the amide-III band was calculated within the frequency range of 1220–1291 cm^–1^, the area of the C–H deformation was calculated between 1395–1486 cm^–1^, and the spectral variance of the bend-libration was calculated within the frequency range of 1900–2450 cm^–1^. Fits to kinetics were performed using the Global Fit function in Igor Pro 9.02.

### Fluorescence Recovery after Photobleaching

Droplets were prepared as previously described with the addition of 15 nM Alexa Fluor 532-labeled TDP-43_CTD_ and 1.5 μM ThT. ThT was added to verify development of amyloid structure upon aging. Samples were imaged on a FLUOVIEW FV4000 confocal laser scanning unit (Evident) on an IX83 inverted microscope frame equipped with a 60× oil objective (UPLXAPO60XO, Evident). FRAP image sequences were acquired using a 561 nm laser at 1% power and a detection range of 570–620 nm. Photobleaching was achieved using 100% laser power. Images were acquired over a 10.6 × 10.6 μm area at a pixel spacing of 0.166 μm and a sampling speed of 4.0 μs/pixel. Frames were captured at 2-s intervals with 6 prebleach frames, a 1-s bleaching time and 151 recovery frames.

## Supplementary Material



## Data Availability

All data have been deposited in Figshare at 10.25444/nhlbi.31298437.

## Notes

Full Raman spectra, additional DIC images, representative *z*-scan of a single condensate, ThT fluorescence images, second set of condensate maps at different aging times, spectra and kinetics associated with the water bend-libration, and Raman difference spectra (PDF)
