# Supplementary material for: Kinetics and Spatial Distribution of β‑Sheet Development in TDP‑43CTD Condensate Maturation
Source: ACS Chem Neurosci. 2026 May 22;17(11):2219–28. doi: 10.1021/acschemneuro.6c00226 (PMC13237731; doi:10.1021/acschemneuro.6c00226)
Supplement: Supplementary file 1 [file cn6c00226_si_001.pdf]

Supporting Information

for

Kinetics and spatial distribution of β-sheet development in TDP-43$_{CTD}$ condensate maturation

S. Ramos, M.D. Watson, and J.C. Lee

Figure S1. Representative DIC images showing condensate fusion events

Figure S2. Full Raman spectra for condensates shown in Fig. 1C

Figure S3. Representative *z*-scan of a single condensate

Figure S4. Thioflavin-T fluorescence images of condensates at different aging times

Figure S5. Second set of condensate maps at different aging times

Figure S6. Spectra and kinetics associated with the water bend-libration

Figure S7. DIC images of condensate in **Fig. 5**

Figure S8. Difference spectra of $r_1(t_{39\ h}) - r_1(t_{23\ h})$ and $r_2(t_{39\ h}) - r_2(t_{23\ h})$

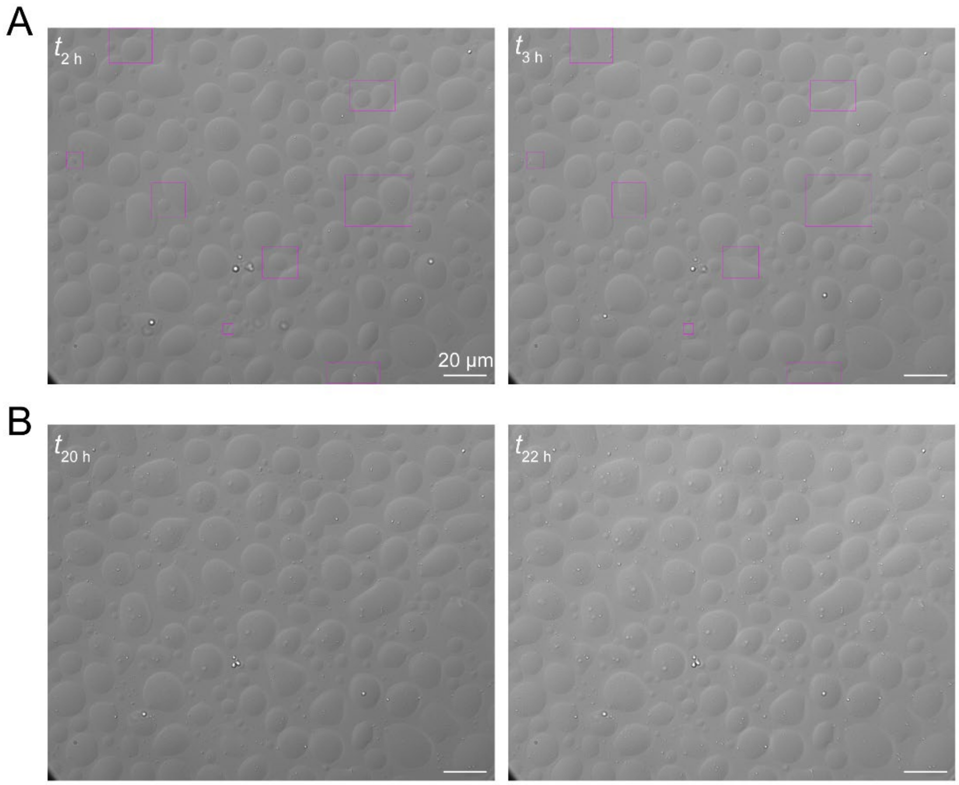

Fig. S1. (A) Representative DIC images of early timepoints showing merging of condensates indicated by magenta boxes, suggesting fluidity. (B) Representative images of later timepoints showing no change in condensate position or size.

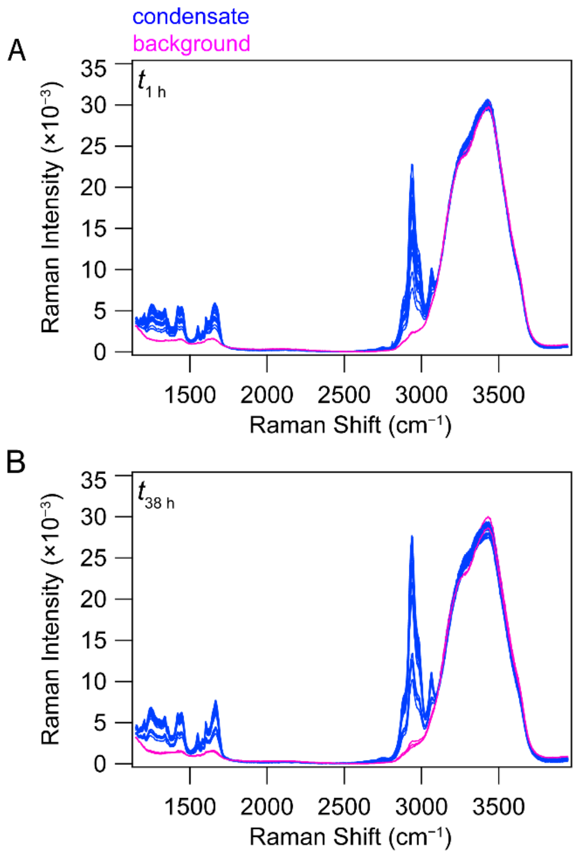

Fig. S2. Full Raman spectra of TDP-43_{CTD} condensates as shown in **Fig. 1C**.

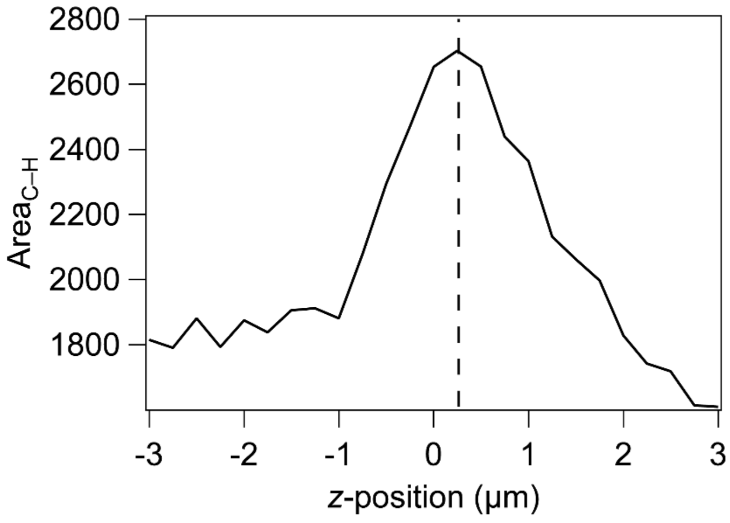

Fig. S3. A representative *z*-scan to find the z-plane for experiments. Z-step sizes are 0.25 μm.

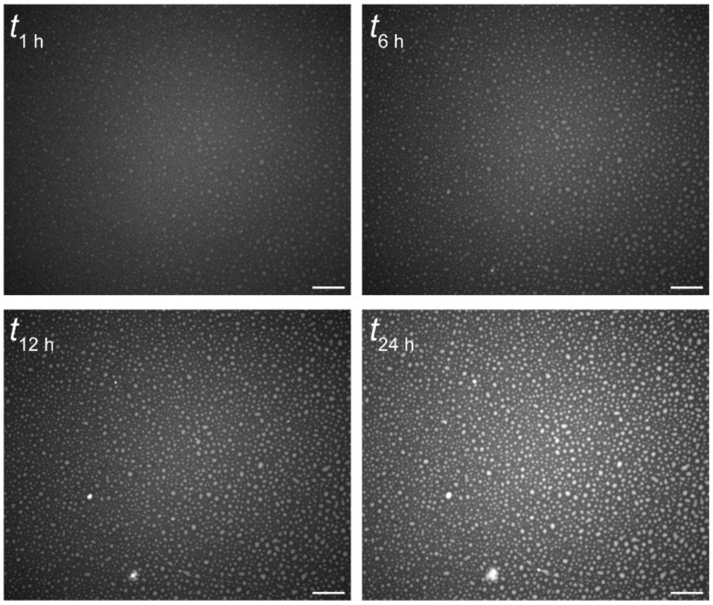

Fig. S4. ThT fluorescence images as a function of aging time. Increases in ThT intensity were observed, suggesting amyloid-like structures within the matured condensates. Widefield fluorescence images were collected on an Evident IX-83 inverted microscope using a 10× objective (Evident UPLFLN10X 0.3 NA), excited by an X-Cite 120LED Boost excitation lamp (Excelitas) using a FF01-433/24-25 excitation filter (Semrock), a FF435-Di01-25x36 dichroic beamsplitter (Semrock). ThT fluorescence was selected by a BLP01-458R-25 emission filter (Semrock) and collected by a Hamamatsu ORCA-Flash4.0 v3 CMOS camera. Scale bars are 100 μm.

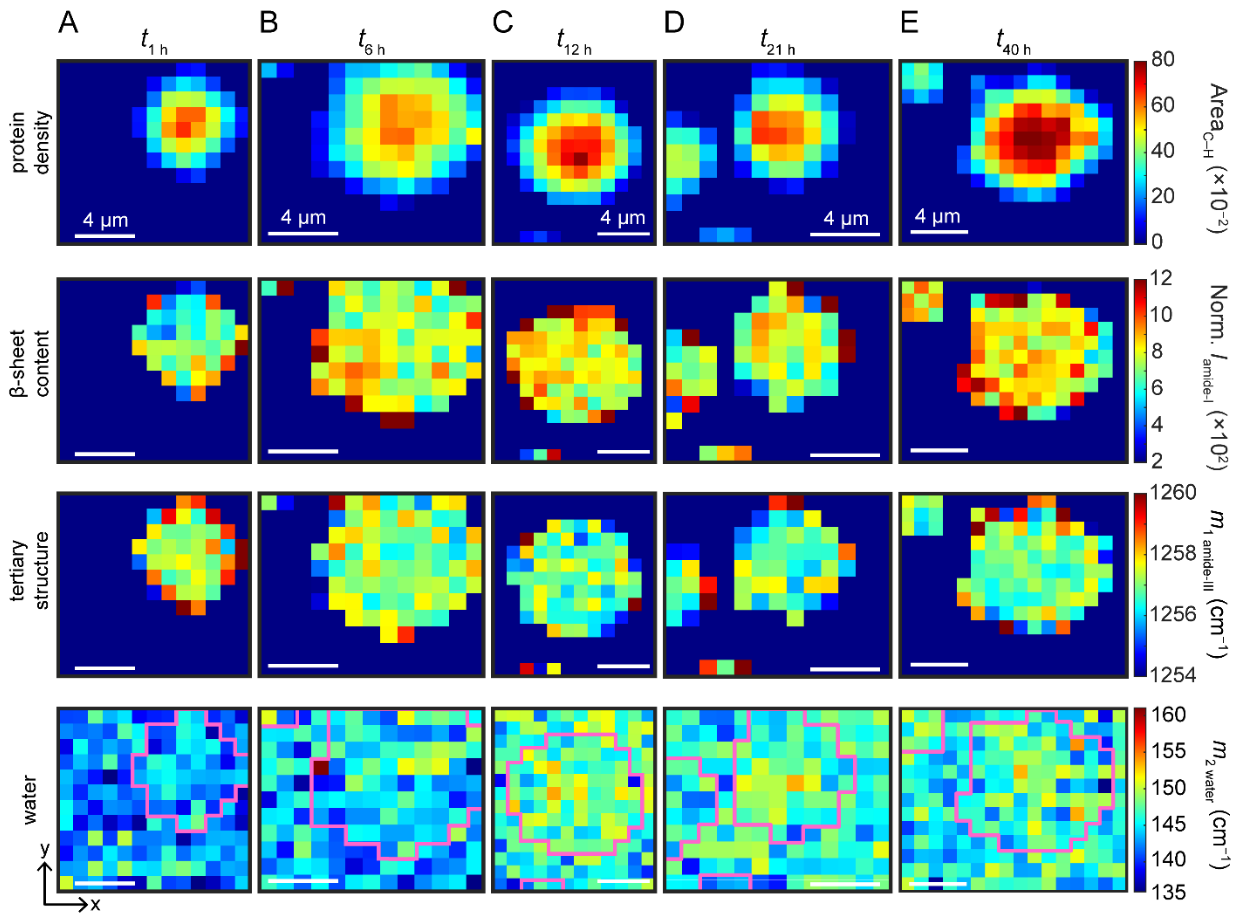

Fig. S5. Raman maps of individual TDP-43$_{CTD}$ condensates. Maps were generated by using the integrated area of the C–H deformation (1395–1486 cm^{-1}) for protein density, the normalized amide-I band intensity at 1669 cm^{-1} for β-sheet content, the weighted mean frequency (first moment, m_1) of the amide-III region (1220–1291 cm^{-1}) for tertiary structure, and the spectral variance (second moment, m_2) of the bend-libration of water (1900–2450 cm^{-1}) at (A) $t_{1\,h}$, (B) $t_{6\,h}$, (C) $t_{12\,h}$, (D) $t_{21\,h}$, and (E) $t_{40\,h}$. Condensate locations are outlined in pink as a guide to the eye (bottom row). Water maps were generated from uncorrected spectra, whereas background-corrected spectra were used to generate the other maps. [CTD] = 15 μM in 10 mM NaPi, 200 mM NaCl, pH 7.4, 20 °C. Step size was 1 μm. Scale bars are 4 μm.

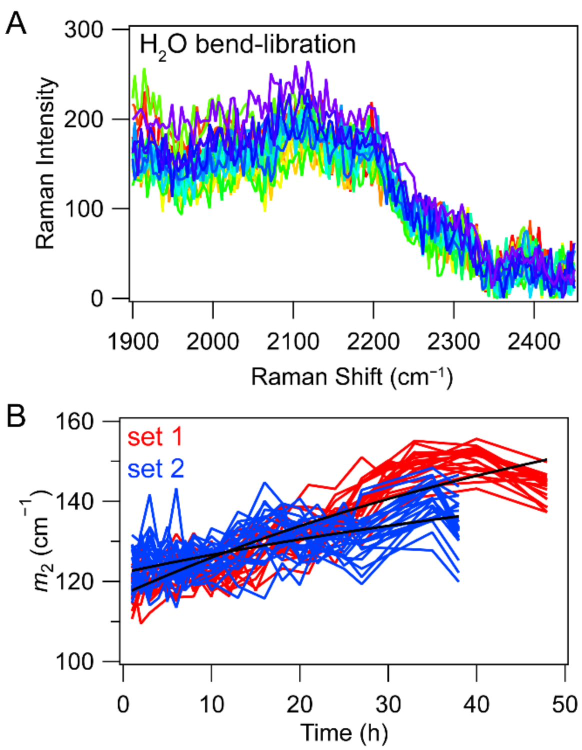

Fig. S6. Analysis of Raman spectral changes associated with the water bend-libration band as a function of TDP-43CTD condensate aging. (A) Representative set of time-dependent spectra within a condensate. A constant offset has been applied. (B) Kinetic traces of the spectral variance (the second moment, m_2) of the water bend-libration (1900–2450 cm^{-1}) for individual condensates ($k = 4.0 \pm 0.3 \times 10^{-6}$ s^{-1}). Two independent data sets are shown in red and blue. Global fits for each set are shown as black lines; rate (k) constant is the average and standard deviation from both sets.

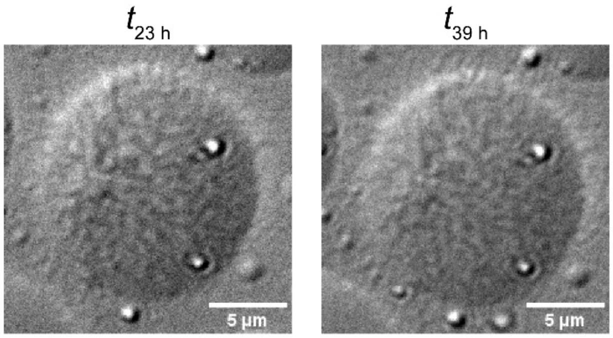

Fig. S7. DIC of condensate with filamentous growth with maps shown in **Fig. 5**. Aging times and scale bars are as indicated.

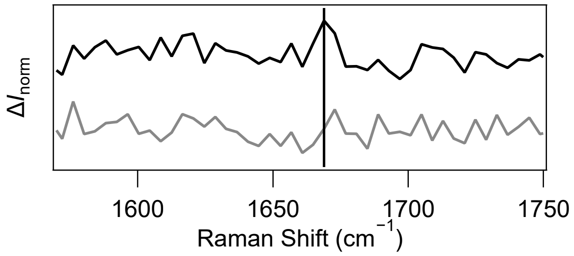

Figure S8. Comparison of difference spectra for each ROI (r) at different times ($r_1(t_{39\ h})-r_1(t_{23\ h})$, black and $r_2(t_{39\ h})-r_2(t_{23\ h})$, gray) in **Fig. 5** showing that β-sheet structure continues to increase at r_1 (black) whereas r_2 (gray) is an area in which the protein secondary structure remains unchanged. Solid line indicates the frequency position of 1669 cm^{-1}.
